# A Text-Book of Diseases of the Nose and Throat

**Published:** 1899-09

**Authors:** 


### *BOOK REVIEWS, PAMPHLETS, EXCHANGES.*

### *BOOK REVIEWS.*

[Contributions solicited for review.]

A TEXT-BOOK OF DISEASES OF THE NOSE AND THROAT. By D. Braden Kyle, M.D. Clinical Professor of Laryngology and Rhinology, Jefferson Medical College; Consulting Laryngologist, Rhinologist and Otologist, St. Agnes Hospital; Bacteriologist to the Philadelphia Orthopedic Hospital and Infirmary for Nervous Diseases; Fellow of the American Laryngological Association, etc., published by W. B. Saunders, Philadelphia. Price $4.00 cloth; $5.00 sheep.

Personal acquaintance with the author of this book has probably biased us somewhat as to its excellency. Dr. Kyle has succeeded in giving to the profession a most excellent work on the diseases of the nose and throat. The work is by no means exhaustive and yet its author has treated in a very clear and concise manner every phase of the subject, giving in the main the most advanced and accepted views.

The colored plates are excellent and works of art, showing the deft artistic hand of Miss Washington in a very pronounced degree. It is very hard for any author to write a book whose views will correspond entirely with every one who is engaged in the same line of work. This cannot be expected. Any book must show its author's individuality in that he will always give such treatment as he is accustomed to follow. This frequently is not in accord with the reader of the book, but taking the work throughout, Dr. Kyle has given to the profession, views and ideas which on the whole are safe to follow. Some subjects are treated of in this work which are not usually found in others or at least not given such a clear and distinct prominence. We refer for instance to Chapter 7, where the author discusses the various kinds of ulcers in the nasal cavities. Every rhinologist has met with these various kinds of ulcers, and frequently he is at a loss to determine their exact etiology. Dr. Kyle, like all other authors of text-books on the throat, is very vague as to the appropriate incision to make in peritonsillar abscess. He seems to have overlooked that anatomical investigation has revealed the distinct presence of a triangular space above and behind the faucial tonsils, and in the majority of cases it is this space which must be punctured if you desire the readiest evacuation. By this means you do not puncture at random.

Then again on the question of the operative removal of adenoids, the author advises the use of Gottstein's curette and the finger nail. Many good men still hold that the bulk of the adenoid growth should be removed first with the forceps and then the curette can be used. What fatalities as have occurred after this operation, have been almost exclusively after the use of the curette.

However, this work of Dr. Kyle is very safe to follow, and we congratulate both him and the publishers on its excellency throughout. ROY.

